# Biological Activity of Extracts from Differently Produced Blueberry Fruits in Inhibiting Proliferation and Inducing Apoptosis of HT-29 Cells

**DOI:** 10.3390/foods11193011

**Published:** 2022-09-28

**Authors:** Ewelina Kiernozek, Piotr Maslak, Ewa Kozlowska, Ingeborga Jarzyna, Dominika Średnicka-Tober, Ewelina Hallmann, Renata Kazimierczak, Nadzieja Drela, Ewa Rembiałkowska

**Affiliations:** 1 Department of Immunology, Institute of Functional Biology and Ecology, Faculty of Biology, University of Warsaw, 02-096 Warsaw, Poland; 2 Department of Ecology and Environmental Conservation, Institute of Environmental Biology, Faculty of Biology, University of Warsaw, 02-096 Warsaw, Poland; 3 Department of Functional and Organic Food, Institute of Human Nutrition Sciences, Warsaw University of Life Sciences, 02-776 Warsaw, Poland

**Keywords:** blueberry fruit extracts, types of cultivation, antiproliferative activity, apoptosis, necrosis, HT-29 cells, 5-Fluorouracil, Erbitux

## Abstract

For several decades, people have been searching for natural substances of plant origin that, when introduced into the diet, could strengthen immunity, have anticancer properties, and support conventional therapy. The development of agriculture with the implementation of various plant cultivation systems, apart from the economic aspect, results in the search for such cultivation conditions that would contribute to obtaining the most beneficial product for health. Therefore, the aim of our research is as follows: (a) to compare the antiproliferative activity and the ability to induce apoptosis of HT-29 cells by extracts from blueberry fruits deriving from different types of cultivation systems (conventional, organic, and biodynamic); (b) to examine whether the interaction of extracts with anticancer drugs used in the treatment of colorectal cancer is influenced by the type of cultivation, and (c) to investigate whether extracts obtained from fruits from subsequent years of cultivation retain the same biological activity. The results of our study are promising but inconclusive. A statistically significant difference occurred in only one of the two years of the study. The greatest inhibition of proliferation is observed for biodynamic cultivation compared to organic cultivation, while the highest levels of apoptosis and necrosis of HT-29 cells are induced by blueberry fruit extracts obtained from organic cultivation. The complementary effect of the extracts on the inhibition of HT-29 cell proliferation by anticancer drugs (5-FU and Erbitux) is not demonstrated. The induction of apoptosis by 5-FU is not enhanced by blueberry extracts, in contrast to necrosis. The level of apoptosis and necrosis induced by Erbitux is potentiated, but no dependence on crop type is shown. Blueberry fruit extracts from two consecutive years of cultivation did not maintain the same activity. A plausible reason for the variability in the composition and biological activity of fruit extracts obtained from two years of cultivation is the varying environmental conditions.

## 1. Introduction

The therapeutic properties of plants have been studied for centuries. It is still the case that plant or fruit extracts are made, their composition is analyzed, and yet research is ongoing, but the results are not always satisfactory. The contribution of plant-derived 8979 compounds to cancer prevention and therapy is continuously investigated [1,2]. The researchers’ concern is not only cancer therapy by using plant-derived compounds but also the prevention of cancer progression by including plant compounds in conventional anticancer treatments.

The International Agency for Research on Cancer reports that colorectal cancer is the third most common cancer type worldwide [3].

Treatments currently used for colorectal cancer may include surgery, radiation, chemotherapy, and targeted therapy. The use of various chemotherapeutics depends on tumour and patient characteristics [4]. Despite such a wide range of therapies, recurrence and mortality rates remain high. Thus, there is a need to develop complementary therapy based on a diet containing isolated plant-derived compounds or their natural mixtures. It is considered that up to 90% of colorectal cancer may be preventable by changes in diet [5]. In this aspect, edible berries have attracted attention, and the study of the activity of their bioactive compounds is conducted in vitro and in vivo models [6,7,8]. The major bioactive compounds present in blueberries are flavonoids (flavonols, myricetin glycosides, quercetin glycosides, kaempferol, anthocyanins), phenolic acids (benzoic and cinnamic acid), tannins (proanthocyanidins), vitamins (C, B, E, A, ascorbic acid), stilbenes (pterostilbene) and others, such as β-carotene, lutein, potassium, calcium, magnesium [7]. Ascorbic acid, anthocyanins, and phenols show high antioxidant potential [9]. The content of antioxidants depends on many factors, among others fruit maturity, field location, farming system, or weather conditions [10,11,12,13]. Researchers involved in the analysis of the chemical composition and therapeutic potential of plant extracts postulate the antitumour effect of blueberry extract on colorectal and other cancers [14,15,16,17,18,19,20,21].

Many plants with a therapeutic effect grow in natural conditions. In turn, the progress in agriculture is aimed at obtaining crops with the most beneficial composition for human health. Various methods of plant cultivation are used in agriculture and horticulture, depending on the environmental approach, economic calculation, and technological possibilities of farmers. The following farming systems are used: (a) conventional—the most common system, based on the use of synthetic pesticides and fertilizers, it is characterized by low biodiversity; (b) agricultural system based on genetically modified organisms (GMOs)—organisms that have acquired desirable and naturally inaccessible traits using biotechnological methods; (c) organic—eliminating from the cultivation all synthetic fertilizers, pesticides, and genetically modified organisms, and focusing on natural farming methods [22], based on the EU legal act [Regulation (EU) 2018/848 on organic production and labelling of organic products and repealing Regulation (EC) No 834/2007]; (d) biodynamic—an additional system of farming based on the anthroposophy holistically treating the agricultural farm as an organism (philosophy of Rudolf Steiner, 1924). This method is very similar to the organic type; however, it implements some specific measures such as biodynamic preparations and a moon calendar. It was created as a response to the devastating impact of synthetic fertilizers and pesticides on the environment and human health [23].

The results of previous research have demonstrated that the following: (a) blueberry dried extracts and fractions, as well as ethanol/water extracts, can have a preventive effect on colon cancer based on their antiproliferative activity on HT-29 and Caco-2 cells [20,24]; (b) blueberry extract demonstrates a positive effect on oxaliplatin [25]; (c) pterostilbene, one of the active components of blueberries, sensitizes colon cancer cells to 5-fluorouracil [26]; (d) the content of biologically active compounds depends on various agents such as farming system, crop location, and weather conditions [10,11,12,13].

Taking into account the above reports, it can be hypothesized as follows: (a) the type of blueberry cultivation results in a difference in the biological activity of blueberry fruit extracts, including anticancer activity, (b) blueberry fruit extracts can act complementarily with the most common chemotherapeutics for colorectal cancer, 5-Fluorouracil (5-FU) and Cetuximab (Erbitux), and their potential to influence depends on the type of crop, and (c) extracts obtained from the cultivation of the following years retain the same biological activity.

Our hypotheses were verified as follows: (a) the antiproliferative and apoptosis-inducing potential of ethanol extracts from blueberry fruits derived from conventional, organic, and biodynamic crops on HT-29 cells was analyzed; (b) the interference of fruit extracts from different crops with 5-FU and Erbitux was examined; (c) the antiproliferative and apoptosis-inducing activity of blueberry fruit extracts derived from two consecutive years was compared. Two-year field studies are the standard for investigating the impact of the agricultural production system on crop quality.

## 2. Materials and Methods

### 2.1. Methods for the Chemical Analysis of Blueberry Fruits

The blueberry (*Vacciniumcorymbosum* L.) cultivation experiment was conducted in two cropping seasons (2016–2017) on private farms with different production systems (conventional, organic, and biodynamic). In each year, fruits were harvested in three different field replicates belonging to a given production system, and the results from these three replicates were then averaged. All samples were frozen at −80 °C and then lyophilized using a Labconco 2.5 freeze-dryer (Labconco Corporation, Kansas City, MO, USA). The freeze-drying process was carried out using the following parameters: temperature −40 °C, pressure 0.100 mBa. After freeze-drying all samples were ground in a laboratory grinder A-11 and stored in scintillation vials at −80 °C before further analysis. The freeze-dried fruits were subjected to chemical analysis as follows.

#### 2.1.1. Polyphenols Analysis

Polyphenols were measured by HPLC method. In total, 100 mg of powdered dried blueberry powder was mixed with 5 mL of 80% methanol and shaken on a Micro-Shaker 326. Next, all samples were extracted in an ultrasonic bath (10 min, 30 °C, 5500 Hz). Next step was centrifugation (10 min, 6000× *g* rpm, 5 °C). The supernatant was carefully collected in a clean plastic tube and centrifuged again (5 min, 12,000× *g* rpm, 0 °C). In total, 1 mL of supernatant was transferred to an HPLC vial and analysed. For polyphenols separation and identification, a Synergi Fusion-RP 80i Phenomenex column (250 × 4.60 mm) was used. The analysis was carried out with the use of Shimadzu equipment as follows: two pumps LC-20AD, controller CBM-20A, column oven SIL-20AC, spectrometer UV/Vis SPD-20 AV). The polyphenols were separated under gradient conditions with a flow rate of 1 mL min^−1^. The following two gradient phases were used: 10% (*v*/*v*) acetonitrile and ultrapure water (phase A) and 55% (*v*/*v*) acetonitrile and ultrapure water (phase B). The phases were acidified by orthophosphoric acid (pH 3.0). The total time of the analysis was 38 min. The phase-time program was as follows: 1.00–22.99 min, 95% phase A and 5% phase B; 23.00–27.99 min, 50% phase A and 50% phase B; 28.00–28.99 min, 80% phase A and 20% phase B; 29.00–38.00 min, 95% phase A and 5% phase B. The wavelengths were 250 nm for flavonoids and 370 nm for phenolic acids. Phenolic compounds were identified using 99.9% pure standards (Sigma-Aldrich, Poznań, Poland). The sum of the identified compounds belonging to each chemical group was then calculated for each fraction separately (polyphenols, flavonoids, and phenolic acids). These values are shown in Table 1.

#### 2.1.2. Anthocyanins Analysis

Anthocyanins were measured by HPLC method. For that purposes two-steps extraction procedure was used. First step was the same as described previously with polyphenols extraction. Next 2.5 mL of supernatant was mixed with 2.5 mL of 10 M hydrochloric acid (HCl) and 5 mL 100% methanol (HPLC gradient). Samples were kept in cooling place thought 10 min. After that 1 mL of sample was used for HPLC analysis. Isocratic phase was used composed of acidified deionized water (with 5% of acetic acid), acetonitrile and methanol (70:10:20). The wavelength was 530 nm. The flow rate was 1.5 mL min^−1^ Anthocyanins were identified using 99.9% pure standards (Sigma-Aldrich, Poznań, Poland). The sum of identified anthocyanins was calculated and this value is given in Table 1.

### 2.2. Blueberry Extracts Preparation for Cell Line Experiments

The fruit extraction procedure was almost analogous to the chemical analysis. The lyophilisate was weighed at a rate of 2 g per 10 mL of 80% ethanol in plastic tubes. The tubes were then vortexed for 20 s for each sample. The tubes were placed in an ultrasonic bath (15 min, 30 °C, 5 kHz). The samples were then centrifuged (14,000× *g* rpm, temperature 2 °C, time 20 min). The supernatant from the tubes was removed and poured into new tubes. The safe concentration of ethanol in the culture medium should not exceed 0.05%. The concentration of blueberry extracts used in all experiments was 125 μg/mL culture medium.

In the figures, the following abbreviations are used to denote the types of farms from which blueberry fruits were obtained for extract production: ORG—organic; BIOD—biodynamic; CONV—conventional.

### 2.3. Anti-Cancer Drugs

In this study the following two drugs being the therapeutic standard in colon cancer have been used: 20 mM 5-Fluorouracil (Sigma-Aldrich, Poznań, Poland) and 20 μg /mL Erbitux (Merck, Kenilworth, NJ, USA).

### 2.4. Cancer Cell Culture

Human Colorectal Adenocarcinoma Cell Line HT-29 (ATCC HTB-38 American Type Culture Collection) was used. HT-29 cell line is a well-established model used to study the biology of human colon cancers, but also in studies focused on food bioavailability and digestion due to epithelial morphology [27,28]. The experiments were conducted with cells that have been cultured by 3–5 passages. According to Chen et al. the karyotypes of HT-29 line were comparable for HT-29 cells along more than 100 passages [29].

HT-29 cells were cultured in 24-well plates at a density of 15 × 10^4^ cells in 1.0 mL culture medium consisting of the following: RPMI 1640 + GlutaMAX medium (Gibco™, Waltham, MA, USA), 200 mM sodium pyruvate, 100/mL penicillin and 100 µg/mL streptomycin (Sigma-Aldrich, Poznań, Poland), and 10% FBS (fetal bovine serum). Standard conditions of culturing were applied as follows: humidified atmosphere, 37 °C, and 5% of CO_2_.

### 2.5. Scheme of the Experiment

**Figure 1:**
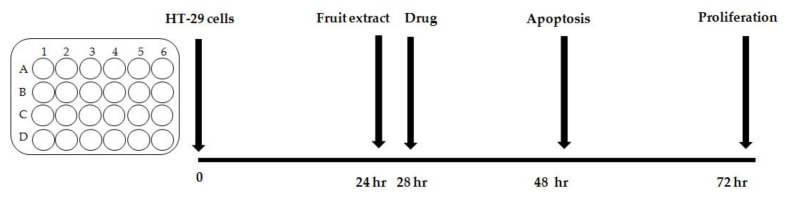
Scheme of the experiment.

The scheme of the experiment is presented in Figure 1. The experiment for each extract was repeated three times. Each culture was performed in triplicate.

Experiment description: The study was modelled on an analogous experiment from a publication on 5-Fluorouracil activity on HT-29 cell lines [30]. Steps of the experiment included the following: (1) seeding of HT-29 cells in 24-well plates; (2) 24-h incubation to allow cell adhesion; (3) addition of the extract; (4) addition of the drug 4 h after the extract in order to simulate the earlier contact of cancer cells with the extract taken as a regular dietary component.; (5) analysis of the level of apoptosis and necrosis 24 h after extract supplementation; (6) proliferation assay 48 h after extract supplementation. Cells from the culture were detached by 5 min incubation with 0.25% trypsin solution (Gibco™).

The results of the blueberry extracts’ activity on the proliferation and level of apoptosis of HT-29 cells were compared to controls as follows: HT-29 cells were incubated in the medium alone and in the medium containing 0.05 % EtOH. Considering that the results for both controls did not differ significantly, the mean value was taken as the numerical value of the control (referred in the figures as CTRL).

### 2.6. Apoptosis Assay

The percentage of apoptotic and necrotic cells was detected by Annexin V/FITC and propidium iodide (PI) staining (BD Pharmingen), according to standard protocol, and analyzed by flow cytometry (BD FACSVerse). Apoptotic and necrotic cells were examined based on AnnV/FITC and PI staining: living cells (AnnV−PI−); early apoptotic cells (AnnV+PI−); late apoptotic cells (AnnV+PI+), and necrotic cells (AnnV−PI+).

### 2.7. Proliferation Assay

To evaluate the proliferation of HT-29 cells, we performed the CFSE assay [31]. Cells were incubated with 20 μM CFSE (Invitrogen) for 10 min at 37 °C and washed three times before culturing. After harvesting, the cells were stained with 7-AAD (BD Pharmingen), which labels dead cells, and then analyzed by flow cytometer BD FACSVerse.

### 2.8. Statistical Analysis

The experiments were performed in two vegetation seasons (2016 and 2017). Three independent extracts were obtained from each type of crop. Each extract was tested in triplicate. Data are presented as means and standard deviations (SD). Statistical significance of differences between the means was evaluated by the t-Student test (or Kruskal–Wallis test in case of non-equal variances). We used one-way ANOVA and then Tukey’s HSD post hoc test if there were three means to compare. Calculations were made in Statistica 13.1 (TIBCO Software Inc., Kraków, Polska). The differences were considered statistically significant at *p* ˂ 0.05.

## 3. Results

### 3.1. The Content of Polyphenols in the Blueberries

Table 1 shows the results of the content of the main polyphenol groups in blueberries from 2016 to 2017. The results are given on a dry weight basis, as freeze-dried blueberries, which are water-free and contain 100 dry weights, were tested.

It should be noted that the standard deviations are high, indicating that there was great variability in the polyphenol content of blueberries from different cultivars of the same type (organic, biodynamic, conventional). This applies in particular to 2017 and to a lesser extent to 2016. This variability can be attributed to environmental factors such as sunlight on the plantation, relief of the land, variability of the soil type, wind protection, etc. This leads to a scattering of results and means that only general differences in the polyphenol content of the fruit can be analyzed.

The results show that organic blueberries (ORG) contained the most total polyphenols in 2016. The same is true for all subgroups of polyphenols tested except for total flavonols, which are highest in biodynamic blueberries (BIOD). Conventional blueberries (CONV) contained more total polyphenols and individual subgroups of polyphenols than biodynamic blueberries in 2016. This is a result contrary to the hypothesis posed at the beginning of the paper.

In 2017, the results were slightly more complicated than in 2016. The content of total polyphenols, total flavonoids and total anthocyanins were highest in BIOD blueberries, while the level of total phenolic acids was highest in ORG blueberries. The level of total flavonoids was almost the same in all types of blueberries. The levels of total polyphenols and all subgroups of polyphenols in CONV blueberries were intermediate between those in ORG and BIOD blueberries.

In summarising, it can be concluded that in both years, blueberries from organic production contained the highest levels of total polyphenols and most subgroups of polyphenols. However, in 2016, this was the case for ORG blueberries, while in 2017, for BIOD blueberries. It can therefore be said that the initial hypothesis was only partially confirmed.

The results also indicate higher levels of polyphenols and their subgroups in 2016 than in 2017. This applies to all compounds tested in ORG and CONV blueberries, as well as to total flavonoids and total flavonols in BIOD blueberries. Possible reasons for the differences in the polyphenol content of the fruit between the years of the study will be given in the discussion of the results.

### 3.2. Effect of Blueberry Extracts on the Proliferative Potential of HT-29 Cells: Activity of Extracts Alone and Interference with Anti-Cancer Drugs

**Figure 2:**
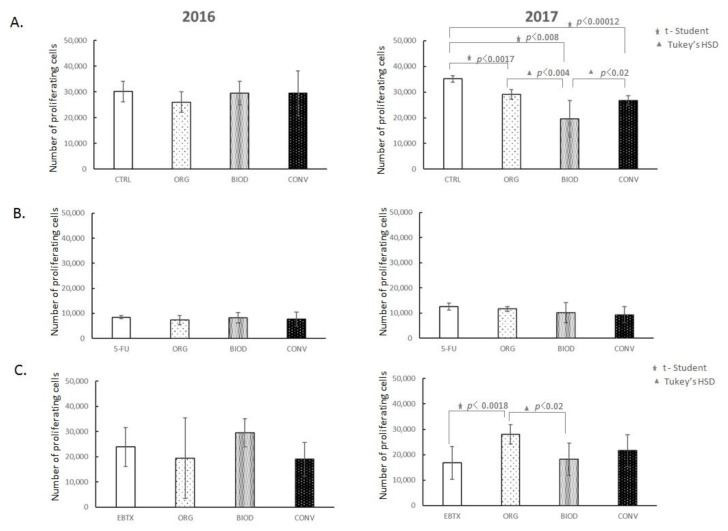
Blueberry extracts influence on the proliferation of HT-29 cells without and with anticancer drugs. (**A**) Effect of blueberry extracts alone; (**B**) Effect of blueberry extracts on the antiproliferative activity of 5-Fluorouracil (5-FU); (**C**) Effect of blueberry extracts on the antiproliferative activity of Erbitux (EBTX). CTRL—HT-29 cell culture without blueberry extracts; ORG, BIOD, CONV—HT-29 cell culture with blueberry extracts from particular types of the production system (ORG: organic, BIOD: biodynamic, CONV: conventional).

The proliferation of HT-29 cells was evaluated after 48 h of incubation with blueberry extracts (Figure 2A). The number of CFSE labelled cells was counted over the same period of time for each sample. The values shown in the figures represent proliferating HT-29 cells among the living population. Blueberry fruit extracts derived from cultivation in 2016 did not affect the level of HT-29 cell proliferation in contrast to extracts from the 2017 year. Extracts from the conventional cultivation-derived fruits showed the greatest decrease in the number of proliferating cells (*p* < 0.00012) compared to the control. Furthermore, we observed statistical differences depending on the type of cultivation. The greatest decrease in the number of proliferating cells was noted for biodynamic cultivation compared to organic (*p* < 0.004) and conventional (*p* < 0.02).

The interference of blueberry extracts on the antiproliferative activity of 5-Fluorouracil (5-FU) is presented in Figure 2B. The 5-FU inhibited the proliferation of colon cancer cells in a dose-dependent manner. The concentration used caused a 3-fold reduction in the number of proliferating HT-29 cells compared to the culture without the drug. We did not notice any influence of blueberry extracts from different types of cultivation on the inhibitory effect of 5-FU.

Cetuximab (Erbitux) is an anti-EGFR monoclonal antibody clinically approved for treating colorectal cancer. Erbitux showed a very weak inhibitory effect on HT-29 cell proliferation. Independently of the type of crop system, blueberry extracts from the 2016 year had no impact on the level of proliferation of HT-29 cells in the presence of the drug (Figure 2C). In contrast, the effect of 2017 extracts showed an inhibitory effect on the drug itself, with the effect of increasing significantly the proliferation level of HT-29 cells only by organic cultivation-derived extracts (*p* < 0.0018).

### 3.3. Effect of Blueberry Extracts on the Induction of Apoptosis and Necrosis of HT-29 Cells: Activity of Extracts Alone and Interference with Anti-Cancer Drugs

**Figure 3:**
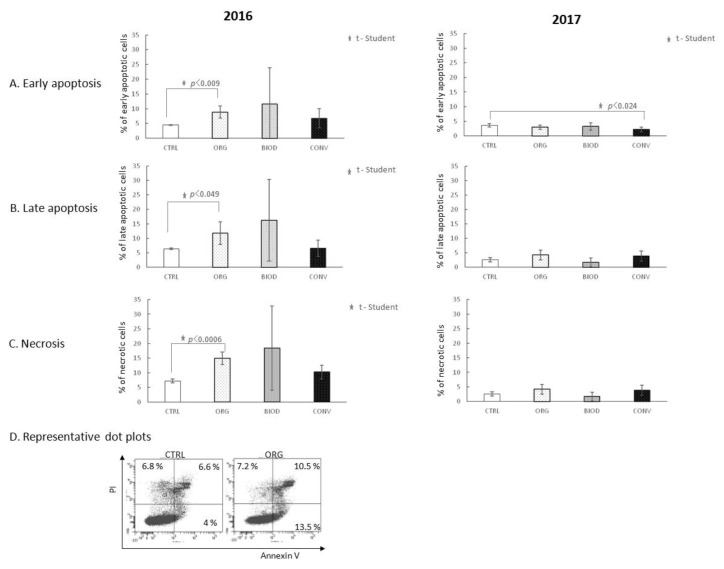
Apoptosis and necrosis-inducing activity of blueberry extracts. (**A**) Effect of blueberry extracts on the early phase of apoptosis; (**B**) effect of blueberry extracts on the late phase of apoptosis; (**C**) effect of blueberry extracts on the induction of necrosis; (**D**) representative dot plots for AnnexinV/FITC and PI staining of the sample showing statistically significant differences. CTRL—HT-29 cell culture without blueberry extracts; ORG, BIOD, CONV—HT-29 cell culture with blueberry extracts from particular types of production system (ORG: organic, BIOD: biodynamic, CONV: conventional).

Blueberry extracts, independently of the year of cultivation, did not affect the early phase of apoptosis of HT-29 cells with the following two exceptions: one case of increase (2016 year, ORG, *p* < 0.009) and one of decrease (2017 year, CONV, *p* < 0.024) of the percentage of early apoptotic cells (Figure 3A). Blueberry extracts from the 2016 organic system increased the percentage of late apoptotic HT-29 cells compared to the control (Figure 3B, *p* < 0.049). The 2017 blueberry extracts did not show a similar effect.

Organic extracts of the 2016 year significantly increased the percentage of necrotic HT-29 cells (*p* < 0.0006) compared to the control (Figure 3C). The 2017 extracts did not demonstrate a necrosis-inducing effect at all.

**Figure 4:**
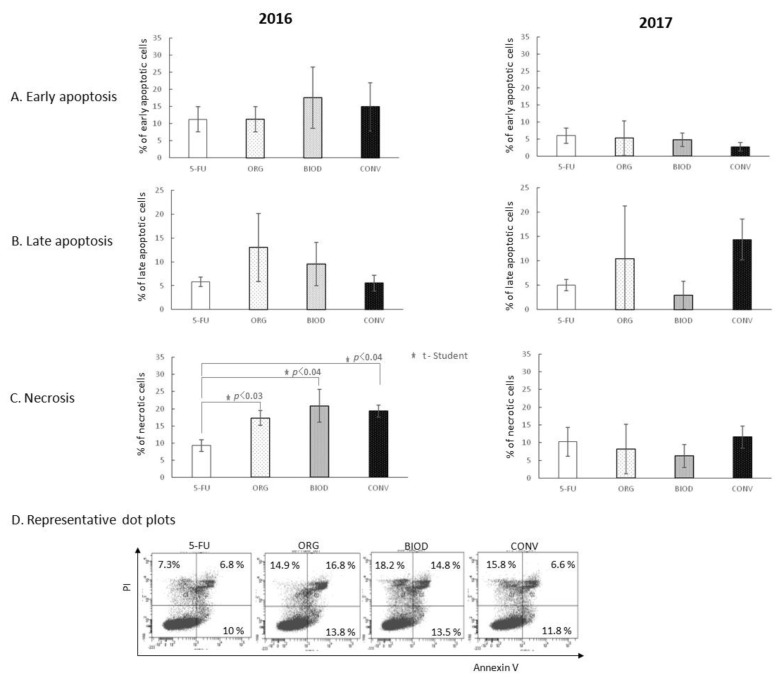
Interference of blueberry extracts on the apoptosis and necrosis-inducing activity of 5-FU. (**A**) Effect of blueberry extracts on the induction of apoptosis by 5-FU at the early phase; (**B**) effect of blueberry extracts on the induction of apoptosis by 5-FU at the late phase; (**C**) effect of blueberry extracts on the necrosis-inducing potential of 5-FU; (**D**) representative dot plots for AnnexinV/FITC and PI staining for 2016. The 5-FU—HT-29 cell culture with 5-FU alone; ORG, BIOD, CONV—HT-29 cell culture with blueberry extracts from particular types of production system (ORG—organic, BIOD—biodynamic, CONV—conventional) and 5-FU.

Not a single blueberry fruit extract interfered with the action of 5-FU in the early as well as the late phase of apoptosis independently of the cultivation year (Figure 4A,B).

The percentage of necrotic HT-29 cells was increased significantly by extracts derived from organic (*p* < 0.03), biodynamic (*p* < 0.04), and conventional fruits (*p* < 0.04) compared to 5-FU alone (Figure 4C). No extract from 2017 showed such activity.

**Figure 5:**
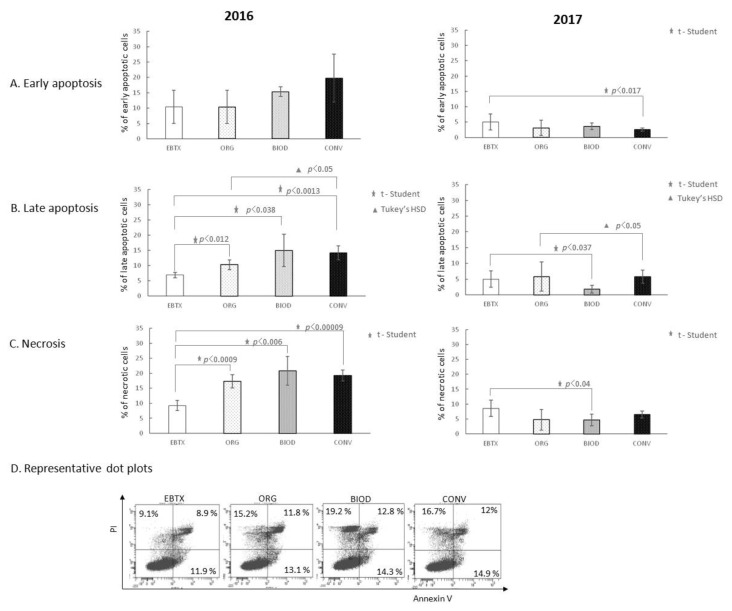
Interference of blueberry extracts on the apoptosis and necrosis-inducing activity of Erbitux. (**A**) Effect of blueberry extracts on the induction of apoptosis by Erbitux at the early phase. (**B**) Effect of blueberry extracts on the induction of apoptosis by Erbitux at the late phase. (**C**) Effect of blueberry extracts on the necrosis-inducing potential of Erbitux. (**D**) Representative dot plots for AnnexinV/FITC and PI staining for 2016. EBTX—HT-29 cell culture with Erbitux alone; ORG, BIOD, CONV—HT-29 cell culture with blueberry extracts from particular types of production system (ORG—organic, BIOD—biodynamic, CONV—conventional) and Erbitux.

We did not observe the interference of blueberry fruit extracts on the level of early apoptosis of HT-29 cells in the presence of Erbitux, with one exception of CONV, resulting in a decrease in the percentage of early apoptotic cells in 2017 (Figure 5A). Blueberry extracts, independently of the type of cultivation in the 2016 year, potentiated the apoptosis-inducing activity of Erbitux and increased the percentage of late apoptotic HT-29 cells compared to Erbitux itself (*p* < 0.012 for organic; *p* < 0.038 for biodynamic; *p* < 0.0013 for conventional cultivation; Figure 5B). However, different effects were noted for the extracts from the 2017 year. Extracts from biodynamic cultivation reduced the apoptotic effect of Erbitux compared to the drug itself (*p* < 0.037; Figure 5B). We noted statistically significant differences between extracts based on fruits from organic and conventional cultivation (*p* < 0.05) both in the 2016 and the 2017 year (Figure 5B).

The level of necrosis of HT-29 cells in the presence of Erbitux was potentiated by all blueberry extracts derived from the 2016 year of cultivation. The percentage of necrotic cells was statistically increased by organic (*p* < 0.0009), biodynamic (*p* < 0.006), and conventional (*p* < 0.00009) system-deriving blueberry extracts compared to Erbitux alone (Figure 5C). Some 2017 extracts showed the opposite or no effect.

As can be seen in most of the figures, the standard deviations are high, which indicates that the biological activity of individual samples of the same group of blueberry extracts is unstable. This instability may result from the high variability in the composition of extracts within the same group, as shown in Table 1.

## 4. Discussion

It has been demonstrated that natural plant-derived compounds can target cancer cells during disease, prevent metastasis, and sensitise them to chemotherapeutic agents [32,33,34]. Colorectal cancer may be preventable by dietary natural compounds influencing carcinogen metabolism [5,6]. Standard treatments used for CRC, including chemotherapy, radiation therapy, surgery, and targeted therapy, are associated with a great number of side effects. In this context, natural fruit or vegetable-deriving compounds may represent a hopeful alternative. Therefore, every aspect of research on agents that could show preventive activity or influence anticancer therapies seems highly justified.

Special attention is focused on berries, which are common worldwide and rich in minerals, vitamins, phenolic acids, and flavonoids [8,35,36]. The preventive, as well as therapeutic activities of berries’ bioactive compounds against colon cancer were extensively investigated in vitro and in vivo in the context of suppression of inflammation, inhibition of proliferation, induction of apoptosis, protection of cells from oxidative damage and inhibition of angiogenesis [37]. A very important aspect of the possible preventive role of natural components of food or extracts is their impact on the intestinal microbiota, and not necessarily on the cancer cells themselves. The composition of bacterial microbiota is considered a significant factor for inflammation-promoted colorectal cancer [38]. It was reported that berries, especially anthocyanin extract, suppressed the pro-inflammatory bacteria in DSS-treated animals [39]. A wide range of experiments on colon cancer prevention were performed with blueberry fruit rich in anthocyanin compounds. The majority of them were based on in vitro models using colon cancer cell lines (HT-29, HCT116, Caco-2) and relied on testing for antiproliferative or apoptosis-inducing activity [20,40,41]. Conflicting results have been obtained in animal studies in which blueberries decreased inflammatory cytokines and pre-cancerous changes in the colon or, on the contrary, were not effective in preventing azoxymethane-induced small intestine and colon cancer [42,43].

A new aspect of research into the anticancer activity of natural plant nutrients is the role of the farming system. Much attention is currently being paid to the potential benefits of organic farming over conventional farming. The hypothesis of a differential antitumour activity of plant-derived nutritional compounds depending on the type of cultivation is based on the differences in the concentration of chemical compounds with documented anti-inflammatory and antioxidant properties in organic vs. conventionally cultivated plants. Hence the idea of research aimed at confirming this hypothesis. Recent studies on the evaluation of the multifactor relationship between agricultural production methods and the physiological effects on rats showed that the production methods affected the composition of the rat feeds, the growth of rats, and their hormonal and immune system status [44,45]. The results of other studies involving a large group of participants showed that a higher frequency of organic food consumption was associated with a reduced risk of cancer [46].

Referring to our first hypothesis that the type of blueberry cultivation results in a difference in the anticancer activity of blueberry fruit extracts, can we conclude that the results of our study are sufficient to verify it? We expected that blueberry extracts of organic and biodynamic fruits would have a stronger antiproliferative potential and would cause the death of HT-29 cells to a greater extent than extracts deriving from conventional plant cultivation. We demonstrated that the antiproliferative activity of blueberry extracts was statistically significant compared to the control (Figure 2A). We showed that the potential to inhibit the proliferation of HT-29 cells was dependent on the type of crop and was the greatest for biodynamic crop-deriving blueberry extracts compared to organic (*p* < 0.004) and conventional (*p* < 0.02) (Figure 2A). However, the biodynamic cultivation was highly unstable, as indicated by the high value of standard deviation. The described biological activity was demonstrated in the case of extracts from 2017. The 2016 extracts showed no antiproliferative activity at all. It must be admitted that we determined the proliferation potential by the number of proliferating HT-29 cells in a living population, which means that all surviving cells proliferated. We can conclude that the proapoptotic and pronecrotic effects of the extracts, discussed below, concerned the 7AAD + cells. The extracts from 2017 caused a decrease in the number of proliferating cells without a visible proapoptotic and necrotic effect, which suggests an influence on the cell cycle rather than a cytotoxic effect.

The level of apoptosis of HT-29 cells in the early, as well as late phase, was higher in the presence of ORG samples prepared from fruits from the 2016 crops (Figure 3A,B). The level of necrosis was also significantly elevated in the presence of ORG blueberry extracts from the 2016 year (Figure 3C). Extracts prepared from blueberry fruits from the 2017 year did not show similar activity. Thus, to some extent, our first hypothesis was confirmed by at least a part of the results. It also turned out that the biological activity of the blueberry extracts does not have to be repeatable in the following years of cultivation.

The results of studies by other authors comparing the biological activity of plant-derived products depending on the type of cultivation seem more decisive. In a study by Olsson et al., organically grown strawberries exhibited stronger antiproliferative activity against cancer colon HT29 and breast MCF-7 cells than conventional fruits [24]. Similar to the previous authors, Kazimierczak et al. have found that naturally fermented beetroot juices from the organically grown beetroots exhibited stronger antiproliferative activity against AGS cells (gastric adenocarcinoma stomach cancer) than the conventional juices [47]. The results of Wojdyło et al. are as ambiguous as ours and point to the diversity of antiproliferative activity of red currant extracts even within the same type of cultivation [48]. According to the literature search, there is, till now, only one study comparing the biodynamic vs. conventional extracts in terms of antiproliferative activity. D’Evoli et al. have proved that crude extract of biodynamic strawberries effectively increases antiproliferative activity against Caco-2 compared to conventional strawberry samples [49].

The next aspect of the study derives from the fact that plant-derived dietary compounds are taken into account in the prevention or complementary treatment of cancer. Hence the idea to verify the second hypothesis that blueberry fruit extracts can act complementarily with the most common chemotherapeutics for colorectal cancer, 5-Fluorouracil (5-FU) and Cetuximab (Erbitux), and their potential to influence depends on the type of crop. We expected that the extracts obtained from BIOD and ORG crops would have the strongest effect in supporting the action of the drugs. The activity of the potent proliferation inhibitory drug, 5-FU, was not modulated by prior exposure of HT-29 cells to blueberry extracts regardless of the year of cultivation (Figure 2B). The 2016 extracts had no effect on the weak antiproliferative effect of the second drug, Erbitux, or, unexpectedly, showed a proliferation stimulating effect, as in the case of 2017 extracts (Figure 2B). A statistically significant stimulatory effect was shown for organic crop-deriving extracts. All extracts from 2016, regardless of the type of cultivation system, increased the level of 5-FU-induced necrosis of HT-29 cells (Figure 4). A similar effect of blueberry extracts from the 2017 crops has not been demonstrated. The effect of Erbitux was not modulated by blueberry extracts obtained from the 2016 crops in the early phase of apoptosis; however, the percentage of HT-29 apoptotic cells induced by this drug increased in the late phase of apoptosis, although no dependence on the type of crop was found (Figure 5). The necrosis-induced activity of Erbitux was potentiated by blueberry extracts in the 2016 year, independently of the cultivation system. Moreover, in this case, a similar effect of blueberry extracts from the 2017 crops has not been demonstrated. On the contrary, there have been occasional, rather random cases of inhibition of Erbitux-induced apoptosis or necrosis by extracts from the 2017 crops. Therefore, in the case of the second hypothesis, we also obtained its partial confirmation.

The third and last hypothesis assumes that the extracts obtained from crops in the following years retain the same biological activity. The research results presented above contradict this hypothesis.

Can we explain the differences in the biological activity of blueberry extracts by differences in the content of selected bioactive compounds? The analysis of the content of polyphenols in mg/100 g DW was performed (Table 1). In this work, we do not present a correlation between the chemical composition of fruit and their biological activity because we are of the opinion that it is not the individual chemical compounds in the fruit that affect cancer cells (or the human organism in general), but rather the composition and proportions of various bioactive substances that have a combined effect on the living organism or isolated cell. Therefore, it was not our intention to make a detailed correlation between the content of individual bioactive compounds and the response of cancer cells to the extracts. For the purpose of this research, attention was paid only to the selected compounds with the highest biological activity described in the literature [7] (Table 1). The numbers show the average content of selected compounds in blueberry fruit lyophilisates. There is a visible lack of stability in the content of chemical compounds in fruit derived from subsequent years, and the values of SD indicate greater differentiation of crops in 2017. Clear differences in the content of particular compounds characterise ORG fruit from 2016. The content of most of the bioactive compounds is greater than in BIOD and CONV fruit. They did not show antiproliferative activity but induced apoptosis and necrosis of HT-29 cells. The concentration of bioactive compounds in ORG fruit was higher than in 2017. The content of particular compounds in fruit from 2017 did not differ significantly depending on the type of cultivation system. In contrast to the 2016 fruit, they showed antiproliferative activity but had no effect on the level of apoptosis and necrosis.

It should be emphasised that the biodynamic method is de facto an organic method, supplemented by additional practices. The results of studies comparing the concentration of bioactive compounds in fruit from biodynamic and conventional agriculture are inconsistent. According to a review paper by Brock et al. [50], many studies confirm the higher abundance of biocompounds in biodynamic crops vs. organic and conventional ones [51,52,53], but at the same time, a number of studies do not confirm this [54,55,56].

The study by Maciel et al. found the highest level of polyphenols in organic mangoes, while lower levels were found in biodynamic and conventional mangoes. On the other hand, d’Evoli et al. found the level of phenolic acids in biodynamic tomatoes to be higher than in fruit from the organic and conventionally grown systems in only one year of the three-year study [55,56]. The above-described inconsistency of the results also occurred in the presented study with blueberries. The reasons for these inconsistencies may be, inter alia, related to the influence of the weather on the development of the tested fruits. According to the climatic portal [57], June 2016 was significantly hotter, drier, and sunnier than June 2017. This is the period of growth and ripening of fruit. In July in 2016—the period of picking fruit—was in turn somewhat more rainy and less sunny than July in 2017. These differences can partly explain the different composition of fruit in 2016 and 2017.

What can we learn from the results of the study of the interaction of extracts and drugs? Currently, 5-Fluorouracil (5-FU) is one of the most effective drugs for early-stage colorectal cancer. Its antitumour effect results from the inhibition of thymidylate synthase, which is essential for DNA replication, which in turn leads to DNA damage, S-phase arrest, and apoptosis [58,59]. Cetuximab (marketed under the name Erbitux) is a chimeric monoclonal antibody that inhibits the epidermal growth factor receptor and blocks a variety of processes in cell biology, including proliferation, survival, and differentiation [60,61]. The use of the extracts in the culture of HT-29 cells prior to the addition of drugs does not show the same pattern of action. It depends rather on the mechanisms of action of the drug itself (Figure 4 and Figure 5). It can be manifested by the destruction of tumour cells by necrosis, as in the presence of 5-FU in the culture medium (Figure 4), or it may result in cell destruction by apoptosis and necrosis, as in the case of the addition of Erbitux (Figure 5). It may also not affect the level of proliferation, as in the presence of 5-FU (Figure 2B), or stimulate proliferation, as in the case of Erbitux (Figure 2C). The results of our research do not show the benefits of using drugs and blueberry extracts together. The type of plant cultivation from which the extracts are obtained seems to be of secondary importance considering the instability of composition in individual years (Table 1). The concept of the role of diet in cancer prevention is not new, but it is mainly directed at the action of particular groups of chemical compounds of plant origin, and this area of research is characterized by greater repeatability of the results and the possibility of comparing them between laboratories through the use of a specific, broad panel of applied tests [62].

Alternative research focuses on the analysis of the activity of natural dietary components included in whole fruits or vegetables. The pros for whole foods in vivo studies, and extracts in vitro, are the cumulative effects of multiple bioactive phytochemicals. The arguments against point to the uncertainty as to which components and in what proportions are responsible for chemoprevention. Additionally, the composition of natural foods or extracts is subject to changes depending on the type of cultivation and environmental factors independent of human activity. This creates a risk that the biological activity of plant extracts, even from the same type of cultivation, may differ from year to year, so it must be verified each time on the basis of a set of commonly accepted and easy-to-compare tests.

## 5. Conclusions

In summary, we showed that, in general, blueberry extracts influenced the proliferation and induced apoptosis and necrosis of HT-29 cells. The proliferation inhibitory effect was demonstrated for fruit extracts from all types of crops in 2017, but the greatest inhibitory potential was revealed in extracts from biodynamic cultivation. In contrast, in 2016, we found an increase in the percentage of apoptosis and necrosis of HT-29 cells under the influence of blueberry fruit extract obtained from organic cultivation. There was no complementary effect of the extracts on the inhibition of HT-29 cell proliferation by 5-FU and Erbitux. The level of apoptosis induced by 5-FU was not potentiated by blueberry extracts, in contrast to the effect on the percentage of necrotic cells. There was an increase in the level of apoptosis and necrosis induced by Erbitux, although no dependence on crop type was found.

A comparison of the biological activity of blueberry fruit extracts from two consecutive years of cultivation showed that they did not maintain the same activity. In both years of the study, blueberries from organic production contained more total polyphenols and most subgroups of polyphenols than fruit from conventional production. However, in 2016, this was the case for organic blueberries, while in 2017 it was the case for biodynamic blueberries. The influence of environmental conditions, including weather, is the most likely reason for the instability of the composition and biological activity of blueberry extracts.

Overall, the study of the biological activity of natural compounds from plants grown according to specific agricultural systems in the context of human health is important and potentially beneficial, but should be studied on a long-term basis due to unpredictable environmental factors.

## Figures and Tables

**Table 1:** Content of phenolic compounds from different chemical group in experimental blueberry extracts. Mean values ± standard deviation.

	Selected Groups of Compounds (mg/100 g DW)	ORG	BIOD	CONV
2016	Total polyphenols	936.82 ± 19.81	771.00 ± 55.54	864.41 ± 68.66
	Total phenolic acid	127.57 ± 13.18	64.23 ± 8.24	113.04 ± 6.45
	Total flavonoids	809.26 ± 13.39	706.77 ± 56.21	751.36 ± 66.09
	Total flavonols	78.69 ± 10.27	89.11 ± 4.43	75.64 ± 13.74
	Total anthocyanins	730.57 ± 18.41	617.67 ± 55.59	675.72 ± 76.33
2017	Total polyphenols	756.17 ± 129.88	798.11 ± 54.50	760.64 ± 92.59
	Total phenolic acid	107.53 ± 10.16	93.22 ± 10.38	97.95 ± 14.47
	Total flavonoids	648.64 ± 120.98	704.89 ± 46.08	662.70 ± 80.22
	Total flavonols	72.08 ± 5.45	70.58 ± 12.08	71.65 ± 9.46
	Total anthocyanins	576.56 ± 118.16	634.31 ± 36.88	591.05 ± 72.98

DW—dry weight; ORG—organic cultivation; BIOD—biodynamic cultivation; CONV—conventional cultivation.

## Data Availability

The data presented in this study are available on request from the corresponding author.
